# Cortical Visual Impairment in CDKL5 Deficiency Disorder

**DOI:** 10.3389/fneur.2021.805745

**Published:** 2022-01-26

**Authors:** Michela Quintiliani, Daniela Ricci, Maria Petrianni, Simona Leone, Lorenzo Orazi, Filippo Amore, Maria Luigia Gambardella, Ilaria Contaldo, Chiara Veredice, Marco Perulli, Elisa Musto, Eugenio Maria Mercuri, Domenica Immacolata Battaglia

**Affiliations:** ^1^Pediatric Neuropsychiatric Unit, Dipartimento di Salute della Donna e del Bambino e Sanità Pubblica, Fondazione Policlinico Universitario Agostino Gemelli, IRCCS, Rome, Italy; ^2^Dipartimento di Scienze della Vita e Sanità Pubblica, Università Cattolica del Sacro Cuore, Rome, Italy; ^3^National Centre of Services and Research for the Prevention of Blindness and Rehabilitation of Low Vision Patients, IAPB Italia Onlus, Rome, Italy

**Keywords:** cortical visual impairment, CDKL5 deficiency disorder, developmental and epileptic encephalopathies, VEP (visual evoked potential), EEG abnormalities

## Abstract

**Background:**

CDKL5 deficiency disorder (CDD) is a developmental encephalopathy caused by pathogenic variants in the gene cyclin-dependent kinase-like 5. Cerebral visual impairment (CVI) is frequent in patients with CDD. In addition to being recognized as a specific feature of the pathology, it has been suggested that visual impairment may correlate with neurodevelopmental outcome and epilepsy severity, but no systematic behavioral visual assessment has been performed. The aim of our study was to evaluate clinical and electrophysiological profile of CVI in patients with CDD, to correlate various aspects of visual function to neurodevelopmental and epileptic features.

**Methods:**

The study included all patients with CDD from the National Pathology Registry. All patients underwent neurological examination, a disease-specific functional assessment, structured clinical evaluation of visual functions, including pattern reversal visual evoked potential (VEP), and a detailed monitoring of epileptic features, including video-EEG.

**Results:**

All the 11 patients recorded in the CDKL5 national registry, 10 females and one male, age range of 1.5 to 24 years (mean 9, SD 7.7, median 6.5), were enrolled. Visual function is impaired in all patients; in particular, visual fields, visual acuity, contrast sensitivity, and stereopsis were consistently abnormal whereas other aspects, such as fixing and tracking, were relatively preserved. Pattern reversal VEP was abnormal in nearly 80% of our patients. No correlation was found among CVI severity, age, level of psychomotor development, EEG abnormalities, and pathology stages even if an overall less abnormal EEG pattern was more often associated with better visual results.

**Conclusion:**

In conclusion, CVI can be considered as a major feature of CDD with a diffuse involvement in several behavioral and electrophysiological aspects. Larger cohorts will help to better clarify the possible prognostic role of EEG severity in predicting both visual and developmental abnormalities.

## Introduction

CDKL5 deficiency disorder (CDD) is a developmental encephalopathy caused by pathogenic variants in the gene cyclin-dependent kinase-like 5 located on the short arm of the X chromosome (X22p13). The gene product, the CDKL5 protein, is highly expressed in the brain, predominantly in neuronal nuclei and dendrites. It has been found to have a role in cell proliferation, neuronal migration, axonal outgrowth, dendritic morphogenesis, and synapse development and function, also in the adult brain (1–5).

Clinical signs of CDD include early infantile onset refractory epilepsy, hypotonia, developmental intellectual and motor disabilities, cortical visual impairment, sleep disorders, associated with minimal dysmorphic features, and possible gastrointestinal and respiratory signs (6).

Median age of epilepsy onset is 6 weeks, with 90% of the patients having signs by 3 months.

Visual impairment is frequent in patients with CDD. It is mainly characterized by poor eye contact and the absence of visual tracking with normal ophthalmological assessment. Recent observational multicenter clinical studies (6, 7) reported fixation and tracking abnormalities, the presence of nystagmus, detection of roving eye movements (slow conjugated movements, mainly horizontal, of the eyes, similar to those observed in sleep), and abnormal optokinetic nystagmus. These findings were extrapolated by parental report of patient's functional abilities, neurologist's physical examination findings, and ophthalmological assessment. The impairment of at least one of these aspects was found in 76% of patients (6). The authors also found a direct correlation between visual deficit and neurodevelopmental outcome (6, 7). Because of this, visual impairment has been proposed as a marker of clinical severity or prognosis even if the pathophysiological mechanisms underlying this deficit in CDD are not clearly understood. A recent study on mouse model of CDKL5 disorder using pattern reversal intracortical visual evoked potential (VEP) reported a dramatic impairment of the cortical response in both juvenile and adult mice (8). The severity of reduction in VEP amplitude was related to the level of visual acuity and contrast sensitivity. The same group analyzed the morphology of the visual pathway from the retina to the primary visual cortex (V1) in CDKL5 null mice. They found reduced density and altered morphology of spines and excitatory synapsis of dorsal lateral geniculate nucleus and V1, but no anomalies in the anterior circuitry from the retina. The abnormal findings in the brain also suggest that there may be a common pathway with other clinical signs of central nervous system involvement and that other electrophysiological techniques, such as EEG, may be used to establish a correlation between brain electrical activity and cortical visual impairment.

It has been suggested (9) that visual impairment may correlate with the three epilepsy stages described in CDD: (I) early onset epilepsy, characterized by daily and polymorphous seizures, associated with a normal to destructured electrical background activity with or without focal anomalies; (II) epileptic encephalopathy; and (III) refractory multifocal and myoclonic epilepsy with destructured EEG and florid multifocal anomalies in wakefulness and sleep (10–13). In particular, a delay in maturation of visual abilities has been described in stage I whereas a regression of some aspects of visual function, such as visual attention, has been described at the beginning of stage II.

Because of the difficulties in obtaining reliable visual assessments in CDD children, no systematic behavioral visual assessment has been performed in correlation of epileptic features.

The aim of our study was to evaluate clinical and electrophysiological profile of CVI in patients with CDKL5-deficient encephalopathy to correlate various aspects of visual function and VEP and to establish whether both the clinical signs and the cortical responses are related to the severity of the clinical and EEG signs of epilepsy.

## Materials and Methods

The study includes patients who diagnosed with CDD identified through the “CDKL5 together toward the cure” association, as part of a project aimed to create a National Register of CDKL5 deficiency disorder. All patients in the registry were contacted. All families agreed to be a part of the study and signed an informed consent. The study was approved by the Ethics Committee of our institution.

Patients were assessed at the Child Neurology Unit and at the National Center of Services and Research for the Prevention of Blindness and Visual Rehabilitation of Visually Impaired, of the University Hospital “Fondazione Policlinico A. Gemelli IRCSS” in Rome.

All patients underwent neurological examination, a disease-specific functional assessment, structured clinical evaluation of visual functions, including pattern reversal VEP, and a detailed monitoring of epileptic features, including video-EEG.

### Disease-Specific Functional Assessment

All patients were scored using the CDKL5 Development Score, a functional scale proposed by Demarest et al. (7) which provides a score from 0 to 7, obtained by adding the stages of psychomotor development reached by the patient (autonomous sitting posture, autonomous standing posture, autonomous walking, rake grip, gripper grip, lallation, and use of single words).

### Assessment of Visual Function

This included an ophthalmological assessment, and a battery of tests assessing various aspects of visual function: fixation, saccades, acuity, visual fields, and attention at distance, was used, adding other aspects of visual function such as contrast sensitivity and stereopsis.

*Ophthalmological assessment* a single pediatric ophthalmologist examined all patients.

Anterior segment examination by handle slit lamp, indirect ophthalmoscopic of the fundus, and cycloplegic refraction by autorefractometry were performed. Myopia was defined as a cycloplegic refraction of −0.5 diopters (D) or less, hyperopia as +2.00D or more, and astigmatism was considered if more than 0.75D.

Slit lamp examination was performed searching for unrecorded alterations and to check lens transparency. Cycloplegic was performed 40 min after administration of tropicamide 1% mydriatic eye drops (1 drop for two times in 15 min) by means of Retinomax 3 Plus Handle Refractometer (Nikon). Fundus examination by indirect opthtalmoscopy with +28 and +20 diopters lens was performed. Fundus abnormalities (i.e., the presence of macular dystrophies or optic nerve alterations) were recorded.

Ocular motility was also observed, and the presence of nystagmus, strabismus, or abnormal ocular movements was recorded.

*Fixation:* The ability to fix was assessed by observing the ability of the infant to fix on a high-contrast target (black/white or colored) target. Fixation is stable if it lasts 3 s, is unstable if it is shorter, and is absent if it is not possible to elicitate.

*Tracking or visual pursuit* was assessed by observing the ability of the infant to follow a high-contrast target (black/white or colored) horizontally, vertically, and in a full circle. Tracking is considered complete if it covers the whole arc, incomplete if it goes for more than 50% of it, brief if it is less than 50% of it, and absent if it cannot be elicitate. For visual pursuit, in addition to quality, its presence in the three different arches was considered.

*Saccadic movements* were assessed using one target per hand. Child's attention was alternatively drawn on the targets, horizontally (right and left) and vertically (up and down). The item was repeated two times each side, noting if infant needed to move the head and did not move only the eyes.

*Acuity* was assessed binocularly by means of the Teller Acuity Card procedure (14–16). This method is based on an inborn preference for a pattern (black and white gratings of decreasing stripe widths depicted on cards) over a uniform field. The location of the left or right position of the test stimulus varies randomly. An observer judges the infant's reaction to the location of the test stimulus based on eye and head movements. The threshold of acuity is taken as the minimum stripe width to which the subject consistently responds. Acuity values were expressed in minutes of arc (or cycles per degree) and were compared with age-specific normative data reported in the literature (17, 18).

*Attention at distance* was tested by moving a colored toy (about 8–10 x 8–10 cm) backward in a small arc away from the child. The maximum distance at which the child still keeps attention on the toy is recorded (19).

*Binocular visual fields* were assessed using kinetic perimetry, according to the technique described in detail by van Hof-van Duin (20). The apparatus consists of two 4-cm wide black metal strips, mounted perpendicularly to each other and bent to form 2 arcs, each with a radius of 40 cm. The perimeter is placed in front of a black curtain, concealing the observer, who can watch the infant's eye and head movements through a peephole. The child is held sitting or lying in the center of the arc perimeter, with the chin supported. During central fixation of a 6° diameter white ball, an identical target is moved from the periphery toward the fixation point, along with one of the arcs of the perimeter, at a velocity of about 3°/s. Eye and head movements toward the peripheral ball are used to estimate the outline of the visual fields. Age-specific normative data for full-term and preterm infants are available (18, 21).

*Contrast sensitivity* was assessed using the Hiding Heidi test. It consists in four cards, one white and the other three with the image of a face on both sides with contrast reducing from 100 to 25%, 10, 5, 2.5, and 1.25%. The picture is presented by moving both the picture and the white card with the same speed, usually horizontally. The side the child looks is noted as response. The level of contrast sensitivity consists in the less-contrasted picture the child looks at.

*Stereopsis* was assessed using the Frisby stereotest. This test is used to assess stereovision at closer distances, requiring eye convergence (22, 23). The participant's task is to detect a circle containing a pattern of geometric objects (the target) visible within a mosaic of similar geometric shapes. The target and background are printed on opposite sides of a Perspex plate and so differ in their physical depth. The angular disparity depends on the thickness of the plate and the distance from the observer.

### Pattern Reversal VEP

Patients were evaluated using the classic pattern reversal VEP protocol (24) with black and white checkerboard presented by a rectangular LED flat-screen monitor (4:3). The stimulation parameters were as follows: luminance 50 candles ^*^ m-2; contrast > 80%. The chess shape was square, and regarding the chess size, three different measures were used in three different stimulation sessions: 1, 0.5, and 0.25 cm, with patient placed 57.3 cm from the screen to obtain angles of visual field of 60', 30', and 15', respectively. The phase change between black and white occurred without changes in screen luminance. The reversal rate pattern has been set at 2 reversals per second. Stimuli released for each test were 100 in total. In total, 4 recording electrodes (Oz, PO7, PO8, and Fz) were placed on the scalp, with reference electrode on Fz and ground electrode on the left ear lobe (A1). Copper disc electrodes were used. The electrode impedance was <3000Ω. The signal was filtered with a 1–250 Hz passband; 50 Hz notch filter active. For each stimulation, activity on the scalp was recorded between 50 ms before and 450 ms after stimulus release. The sampling rate was 4096 Hz. Three sessions were performed at a distance of 10 min from each other using different angles of visual field underlying the check. For each derivation on the scalp, the average of traces obtained after frequent and deviant stimulus was carried out. Potentials > 70 μV have been automatically excluded from the average. For data analysis, amplitudes and latencies of N75, P100, and N145 were evaluated and compared with normative data (25, 26).

### Epilepsy

Epilepsy was classified according to Bahi-Buisson stages (9): (I) early onset epilepsy, characterized by daily and polymorphous seizures, associated with a normal to destructured electrical background activity with or without focal anomalies; (II) epileptic encephalopathy; and (III) refractory multifocal and myoclonic epilepsy with florid multifocal anomalies in wake and sleep. Details on onset of the seizures, progression, and pharmacological therapy were also collected.

### Video-EEG

Patients underwent standard video-EEG during wakefulness and sleep. The recording was made through preassembled caps with 21 electrodes according to the International System 10–20. The EEG lasted about an hour and included, as activation tests, the intermittent photic stimulation (IPS). For IPS, a LED photic stimulator was used. The lamp was placed 30 cm in front of the nasion of the patient. White light flashes had an intensity of about 1 Joule (27). Due to poor patient cooperation, they were not expected to close their eyes during IPS. For the same reason, only increasing frequency protocol was performed (1–50 Hz). Each stimulation lasted 10 s with pauses of 10 s. In case of photoparoxysmal response, the protocol provided for the interruption of IPS. Once the parental consent was obtained, the repetition of IPS was provided at the end of the recording, starting from 50 Hz and decreasing to the frequency at which the photoparoxysmal response was observed.

Data were analyzed through the SystemView Micromed system, and a qualitative analysis of background activity in wakefulness and sleep was carried out. Any paroxysmal anomalies and characteristics of recorded electroclinical episodes were also examined. According to EEG features, the examination was classified as normal EEG; normal background activity and the presence of focal anomalies; abnormal background activity and the presence of focal anomalies; abnormal background activity and the presence of focal and generalized anomalies; and hypsarhythmia. Furthermore, according to seizures type and frequency and the results of EEG examination, the stage of pathology has been established for each patient, according to the classification of Bahi-Buisson et al. (9).

Other information included gender, family history of seizures or other diseases, genetic and imaging studies, and possible comorbidities.

For statistical analysis, continuous variables were expressed in means and standard deviations. Categorical variables were presented as frequencies and percentages. Due to the low sample number, only linear regression was performed to identify a possible correlation between the severity of the visual impairment and age, CDKL5 Development Score, and disease stage.

## Results

All the 11 patients in the CDKL5 national registry, 10 women and one men, age range of 1.5 to 24 years (mean 9, SD 7.7, median 6.5), were enrolled. All patients presented the minimum diagnostic criteria proposed by Olson et al. in 2019 (pathogenetic variants in the CDKL5 gene, severe global psychomotor retardation, and epilepsy onset in the first year of life).

All patients underwent a complete assessment.

### Disease-Specific Functional Assessment

Only one patient was able to walk independently and used single words to communicate. Two patients had achieved the ability to sit and stand independently, but not to walk, mature grip, and use of single words. The other subjects had more impaired motor and verbal functions. Details of the CDKL5 Developmental Scale are shown in Table 1.

**Table 1:** Detailed results for each subject.

**ID**	**Age (y)**	**CDKL5 gene mutation**	**Visual functions**															
			**EYE MOV**	**Fix**	**Tracking**			**Attention at distance**	**Visual Acuity**	**Visual Fields**	**Constrast sensitivity**	**Visual Attention**	**Stereopsis**	**Saccades**	**Pattern VEPs**	**CDKL5 Dev Scale**	**CDD stage**	**EEG**
				**Hor**	**Ver**	**Circle**												
1	1.5	c.1648C>T	Strabismus + roving movements upwards	Unstable	Complete	Complete	Complete	2m	<1/20	Bilateral reduction	25%	Absent	No reaction	No reaction	UT	2	II	Hypsarrhythmia
2	1.7	dup and del Xp22.13	strabismus	Stable	Complete	Uncomplete	Absent	50cm	<1/20	No reaction	No reaction	Absent	No reaction	No reaction	ABN	3	II	Abn background act + multifocal and generalized abn
3	3.5	c.433_ 433delC	Strabismus + nystagmus	Unstable	Brief	Brief	Absent	60cm	<1/20	No reaction	No reaction	Absent	No reaction	No reaction	Absent	4	II	Hypsarrhythmia
4	5.8	c.528G>A	strabismus	Stable	Complete	Uncomplete	Absent	1m	<1/20	Bilateral reduction	25%	Only NC	No reaction	No reaction	Absent	2	II	Hypsarrhythmia
5	6.3	c.7441 + G>C	strabismus	Stable	Complete	Uncomplete	Absent	50cm	<1/20	No reaction	No reaction	Absent	No reaction	No reaction	Absent	3	II	Abn background act + multifocal and generalized abn
^*^6	6.5	del exon 1	Strabismus + roving movements	Brief	Brief	Absent	Absent	10-15cm	UT	No reaction	No reaction	Absent	No reaction	No reaction	UT	0	II	Hypsarrhythmia
7	6.5	del exons 18-21	normal	Stable	Complete	Complete	Absent	1.5m	2/10	No reaction	25%	Absent	No reaction	No reaction	N	3	III	Abn background act + focal abn
8	12	c.533G>A	Strabismus	Brief	Absent	Absent	Absent	10cm	<1/20	No reaction	No reaction	Absent	No reaction	No reaction	Absent	3	III	Abn background act + focal abn
9	16.5	c.587C>T	Strabismus	Unstable	Uncomplete	Absent	Absent	1m	<1/20	No reaction	No reaction	Absent	No reaction	No reaction	N	6	III	Normal background act + focal abn
10	21	del exons 7-8	Strabismus + nystagmus	Unstable	Complete	Uncomplete	Absent	50cm	<1/20	Bilateral reduction	No reaction	Absent	No reaction	No reaction	Absent	6	III	Abn background act + focal abn
11	24	c.645 + G>A	strabismus	Stable	Complete	Complete	Complete	1.5m	3/10	No reaction	5%	Absent	No reaction	No reaction	N	7	III	Normal background act + focal abn

* *male; UT, Untestable; ABN, abnormal; N, normal*.

### Assessment of Visual Function

**Figure 1:**
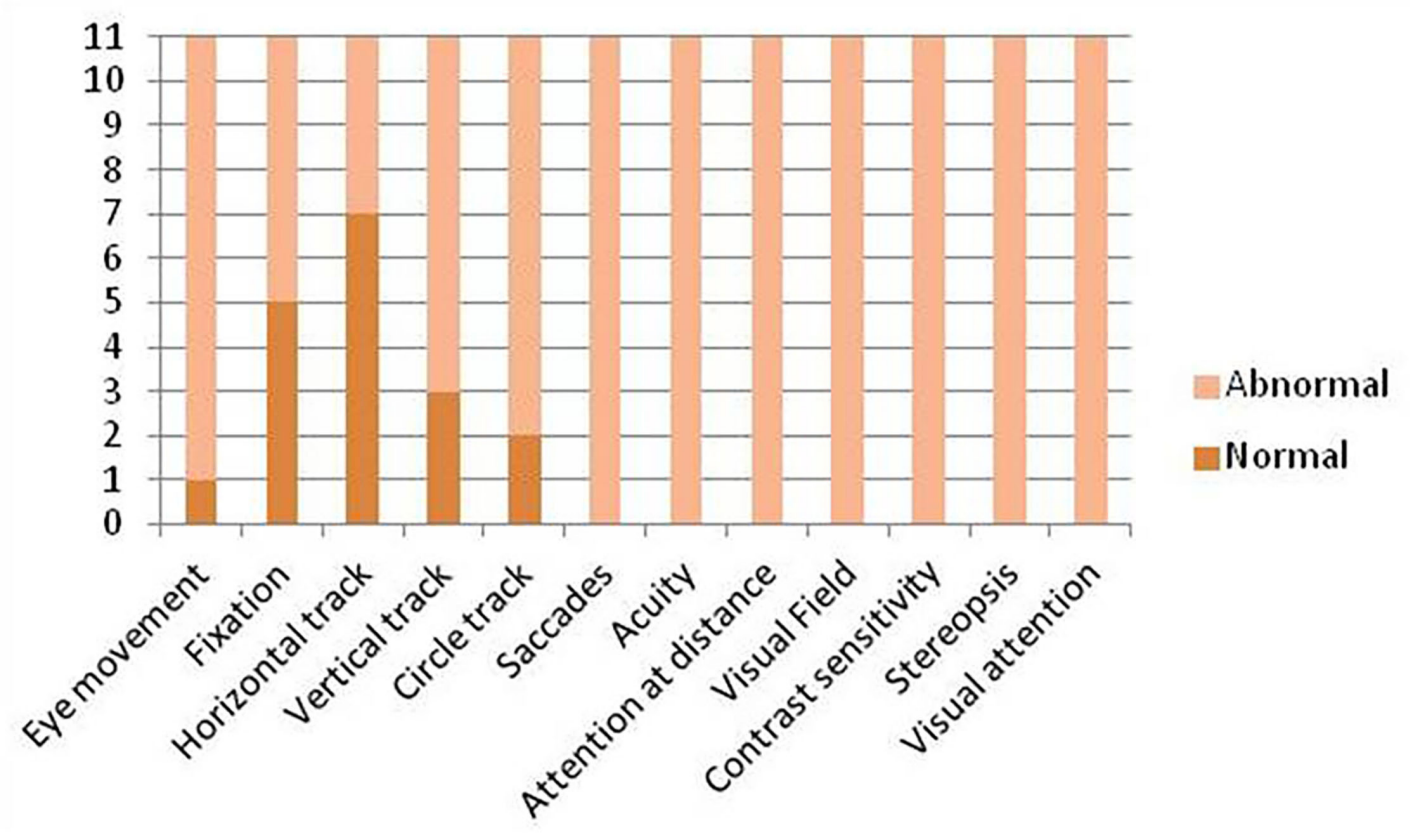
Visual function overview.

All patients completed the assessment and showed clinical characteristics compatible with cerebral visual impairment (Table 1 and Figure 1).

*Ophthalmological assessment* no abnormalities in anterior segment were found, and no media or lens opacity was observed. Optic nerve head slight pallor was observed in all but one patient. No other retinal abnormalities were recorded. Second-level ophthalmological examination (OCT, retinography) was not possible due to lack of cooperation. Hyperopia was found in six patients and myopia in two, associated with mild astigmatism. In the remaining three patients, results were not reliable due to lack of cooperation.

All patients were disturbed by light more than normal showing moderate photophobia.

*Eye movements* were conjugated in one of the 11 patients, and the remaining 10 patients presented strabismus, four esotropia, and six exotropia.

*Fixation* was present and stable in five of the 11 patients, unstable in four, brief in the remaining two patients.

*Tracking or pursuit:* Horizontal tracking was complete in seven out of 11 patients, incomplete in one, brief in two, and absent in the remaining patient.

Vertical tracking was complete in three patients, incomplete in four, brief in one patient, and absent in the remaining three.

Tracking in a circle was complete in two patients and absent in the remaining nine.

*Saccadic movements* were not elicitable in all patients, both horizontally and vertically.

*Visual acuity* was not testable in one out of 11 patients and reduced in the remaining 10 patients.

*Attention at distance* was impaired in all patients, with five of the 11 patients keeping attention for a distance between 1 and 2 m, four patients 50 > 100 cm, and the remaining two patients <50 cm.

The *visual field* was not evaluable in eight patients and restricted in the remaining three patients.

*Contrast sensitivity* was not evaluable in seven patients and reduced in the remaining four.

*Stereopsis* was absent in all patients.

*Visual attention* was absent in 10 of the 11 patients and present only in the noncompetitive modality in the remaining patient.

### Pattern Reversal VEP

In three out of 11 patients, it was possible to obtain a visual evoked potential of normal morphology and latency. In five patients, no response could be elicited, and in one patient, VEP was of low amplitude and altered morphology. The remaining two were too irritable to get reliable potentials due to movement artifact.

### Epilepsy

Six out of 11 patients were in the second stage of epilepsy, the other five in the third. As expected, patients in phase II present daily and polymorphic seizures (spasms, myoclonic, and tonic) and all but one (n ° 4) are on complex antiepileptic drug treatment (two or more drugs). Among the stage III patients, two have daily seizures, 3 every week. All are on complex antiepileptic treatment.

### Video-EEG

No patient had normal EEG, two had recognizable background activity with focal anomalies, three altered background activity and focal anomalies, two altered background activity and multifocal and diffuse anomalies, and four presented with hypsarrhythmia.

None exhibited a photoparoxysmal response during IPS.

### Statistical Analysis

For each patient, the number of impaired areas of visual abilities was used as an independent variable, and a linear regression was performed with age, CDKL5 Development Score, and CDD stage as dependent variables. No significant values of corrected R-squared were obtained for any of the three variables (severity of CVI and age: corrected R-squared = −0,11; severity of CVI and CDKL5 Development Score: corrected R-squared = −0.09; severity of CVI and CDD stage: corrected R-squared = −0,1).

## Discussion

Visual impairment has been reported to be frequent in patients with CDD (28), but the possibility to assess various aspects of visual function was limited by the poor collaboration of these subjects at the time they have to perform structured routinely used visual assessments. Most information on aspects of visual function comes from the ophthalmologic assessment or from caregivers' observation. In this study, using a battery of tests that have been specifically designed for young children with relatively poor collaboration, easy to be performed even in young children with multisensory or cognitive impairment (29–31), we were able to perform a detailed assessment in a cohort with a wide range of age and severity.

This allowed us to use, for the first time, a structured assessment of visual function in combination with a detailed ophthalmological assessment in patients with CDD and with the assessment of evoked potentials. Compared with previous studies, we combined the use of a structured visual assessment and electrophysiological examinations and this consented to define a wider description of visual abilities regardless of severity and pathology stage (6, 7).

Our data confirmed that visual function is impaired in all patients but the possibility to assess different aspects of visual function allowed providing more details on the extent and severity of the involvement.

More cortical aspects of functions, such as visual fields, visual acuity, contrast sensitivity, and stereopsis, which require the integrity of cortical–subcortical networks, were consistently abnormal.

Visual evoked potential was also abnormal in nearly 80% of our patients. These data are in agreement with previous studies assessing visual function in epileptic encephalopathy (30). Animal models of CDKL5 deficit suggest a possible role of an occipital cortical involvement connected to a specific impairment of visual acuity and contrast sensitivity (8, 32).

In contrast, other aspects, such as fixing and tracking, were relatively preserved. These aspects are typically considered to be mediated by subcortical structures but, even if relatively preserved, were still frequently affected in most patients. Although this was not systematically explored in our patients using imaging, the impairment of these aspects is likely to be in relation to the involvement in subcortical areas such as basal ganglia and lateral geniculate nucleus, as described by Mazziotti (8) and Lupori (31) in CDD animal models. It is of interest that while tracking horizontally and, partly, vertically was relatively spared, when increasing the complexity of action, requiring to track in a circle, most of the patients with CDD showed increasing difficulties.

Therefore, our data showed that visual impairment does not appear limited to more mature cortical functions, but may also involve more basic aspects that rely on subcortical structures.

The severity of visual deficit did not appear to be specifically related to age, as severe signs were found not only in infants assessed soon after diagnosis but also in the oldest ones and throughout the whole spectrum of age. Similarly, there was no obvious association between visual function and CDKL5 Development Scores. Interestingly, relatively sparing of these aspects was not always found in patients with less severe scores on the CDD developmental scores probably because these children, even if showing relatively normal tracking, still had severe involvement in all the other visual functions that are important for eye and hand coordination and other developmental aspects.

The relationship between visual function and EEG and disease stage was more complex. We were unable to observe a consistent association between severity of visual function and EEG abnormalities or pathology stages. This is possibly due to the relatively small sample size in our study and to the fact that we did not have any child in stage I. The results were too small, and the number of variables was too high to allow a meaningful analysis. Severe EEG patterns such as hypsarrhythmia were not always associated with the more severe diffuse visual impairment. It should be noted however that, even if did not apply to all the individual cases, a more organized background activity and an overall less abnormal EEG pattern were more often associated with better visual results. This was found in all the three patients with normal pattern reversal VEP, with two of the three also having relatively more preserved aspects of visual function. These findings are partially in agreement with previous studies on animal models (8, 32) reporting that VEP correlated with visual function and more specifically with visual acuity and contrast sensitivity. Similarly, subjects with less abnormal EEG also had better CDKL5 Development Scores.

## Conclusion

In conclusion, CVI can be considered as a major feature of CDD with a diffuse involvement in several behavioral and electrophysiological aspects. None of our patients had a normal profile of visual function and the impairment involved both cortical and subcortical aspects. Larger cohorts with a wider range of EEG abnormalities and disease stages will help to better clarify the possible prognostic role of EEG severity in predicting both visual and developmental abnormalities.

## Data Availability Statement

The raw data supporting the conclusions of this article will be made available by the authors, without undue reservation.

## Ethics Statement

The studies involving human participants were reviewed and approved by Comitato Etico Università Cattolica del Sacro Cuore, Rome, Italy. Written informed consent to participate in this study was provided by the participants' legal guardian/next of kin. Written informed consent was obtained from the minor(s)' legal guardian/next of kin for the publication of any potentially identifiable images or data included in this article.

## Author Contributions

MQ, DR, DB, and EMM contributed to the conception of the subject of the manuscript and wrote and revised the manuscript. DR, MPet, SL, LO, and FA contributed to the visual assessment. MQ, DB, MG, CV, IC, MPer, and EM contributed to clinical and electrophysiology assessment. All authors contributed to the article and approved the submitted version.

## Conflict of Interest

The authors declare that the research was conducted in the absence of any commercial or financial relationships that could be construed as a potential conflict of interest.

## Publisher's Note

All claims expressed in this article are solely those of the authors and do not necessarily represent those of their affiliated organizations, or those of the publisher, the editors and the reviewers. Any product that may be evaluated in this article, or claim that may be made by its manufacturer, is not guaranteed or endorsed by the publisher.
